# Spermatogonial stem cells and progenitors are refractory to reprogramming to pluripotency by the transcription factors *Oct3/4*, *c-Myc, Sox2* and *Klf4*

**DOI:** 10.18632/oncotarget.14327

**Published:** 2016-12-28

**Authors:** Sébastien Corbineau, Bruno Lassalle, Maelle Givelet, Inès Souissi-Sarahoui, Virginie Firlej, Paul Henri Romeo, Isabelle Allemand, Lydia Riou, Pierre Fouchet

**Affiliations:** ^1^ CEA DRF iRCM SCSR, Laboratoire de Recherche sur la réparation et la Transcription dans les cellules Souches, UMR 967, F-92265 Fontenay-aux-Roses, France; ^2^ INSERM, UMR967, F-92265 Fontenay-aux-Roses, France; ^3^ Université Paris Diderot, Sorbonne Paris Cité, UMR 967, F-92265 Fontenay-aux-Roses, France; ^4^ Université Paris Sud, UMR 967, F-92265 Fontenay-aux-Roses, France; ^5^ CEA DRF iRCM SCSR, Laboratoire de Gamétogenèse, Apoptose et Génotoxicité, UMR 967, F-92265 Fontenay-aux-Roses, France; ^6^ CEA DRF iRCM SCSR, Laboratoire de Radiopathologie, UMR 967, F-92265 Fontenay-aux-Roses, France; ^7^ INSERM U1016, Institut Cochin, Paris 75014, France; ^8^ CNRS UMR8104, Paris 75014, France; ^9^ Université Paris Descartes, Sorbonne Paris Cité, Faculté de Médecine, Paris 75014, France

**Keywords:** stem cell, germinal, reprogramming, pluripotency, testis

## Abstract

The male germinal lineage, which is defined as unipotent, produces sperm through spermatogenesis. However, embryonic primordial germ cells and postnatal spermatogonial stem cells (SSCs) can change their fate and convert to pluripotency in culture when they are not controlled by the testicular microenvironment. The mechanisms underlying these reprogramming processes are poorly understood. Testicular germ cell tumors, including teratoma, share some molecular characteristics with pluripotent cells, suggesting that cancer could result from an abnormal differentiation of primordial germ cells or from an abnormal conversion of SCCs to pluripotency in the testis. Here, we investigated whether the somatic reprogramming factors Oct3/4, Sox2, Klf4 and c-Myc (OSKM) could play a role in SSCs reprogramming and induce pluripotency using a doxycycline-inducible transgenic Col1a1-4F2A-OSKM mouse model. We showed that, in contrast to somatic cells, SSCs from adult mice are resistant to this reprogramming strategy, even in combination with small molecules, hypoxia, or p53 deficiency, which were previously described to favour the conversion of somatic cells to pluripotency. This finding suggests that adult SSCs have developed specific mechanisms to repress reprogramming by OSKM factors, contributing to circumvent testicular cancer initiation events.

## INTRODUCTION

In the postnatal testis, spermatogonial stem cells (SSCs) maintain spermatogenesis throughout the mammalian reproductive life. SSCs can self-renew or differentiate into spermatogonial progenitors, which undergo meiosis and eventually differentiate to sperm. The male germinal lineage is considered unipotent because its fate is to produce sperm. However, this lineage appears to exhibit a singular plasticity compared with somatic lineages in its potential to spontaneously reprogram *in vitro* into a pluripotent state [1].

Using mouse models, several authors have described the spontaneous generation of colonies of pluripotent stem cells (ES-like cells) during the long-term *in vitro* culture of SSCs, even though these events are rare [2–4]. In addition, primordial germ cells (PGCs), the embryonic precursors of SSCs that emerge from the epiblast at 6.5 dpc, can also be reprogrammed into pluripotent embryonic germ cells (EGs) when cultured *in vitro* in the presence of specific growth factors or chemical compounds [5]. These PGCs can also produce teratomas after transplantation into the postnatal testis [6]. However, this ability of PGCs to become pluripotent seems to be progressively lost up to 13.5 dpc in embryos. The capacity of germ cells to reprogram is thought to play a role in testicular germ cell tumour initiation [7].

The mechanisms involved in the reprograming of postnatal SSCs remain poorly understood. The dual depletion of *Dmrt1* and *p53* plays a role in the reprogramming of an established culture of SSCs originating from neonatal testis, but the depletion of these genes does not produce pluripotent colonies in CD9-selected SSCs from the pup testis [8, 9]. Yamanaka's transcription factors, *Oct3/4*, *Sox2*, *Klf4* and *c-Myc* (OSKM factors), play a key role in the reprogramming of somatic differentiated cells into induced pluripotent stem cells (iPSCs) [10]. Somatic and germinal lineages may share some molecular pathways for reprogramming to pluripotency. In line with this hypothesis, OSKM factors greatly increase the frequency of the reprogramming of PGCs into a pluripotent state *in vitro* [11]. In addition, the forced expression of Yamanaka factors favours reprogramming in CD9-selected SSCs freshly extracted from the pup testis but cannot induce ESC-like colonies in established culture of SSCs originating from the neonatal testis [12], which has led to contradictory results about the role of OSKM in SSCs reprogramming. Here, using a doxycycline (DOX)-inducible transgenic Col1a1-4F2A-OSKM mouse model [13], we show that SSCs from adult mice are not prone to reprogramming to a pluripotent state by OSKM factors, in contrast to testicular somatic cells, suggesting that different mechanisms induce and/or inhibit reprogramming in postnatal somatic and germinal lineages.

## RESULTS AND DISCUSSION

### Expression of the OSKM transcription factors in SSCs and progenitors

**Figure 1:**
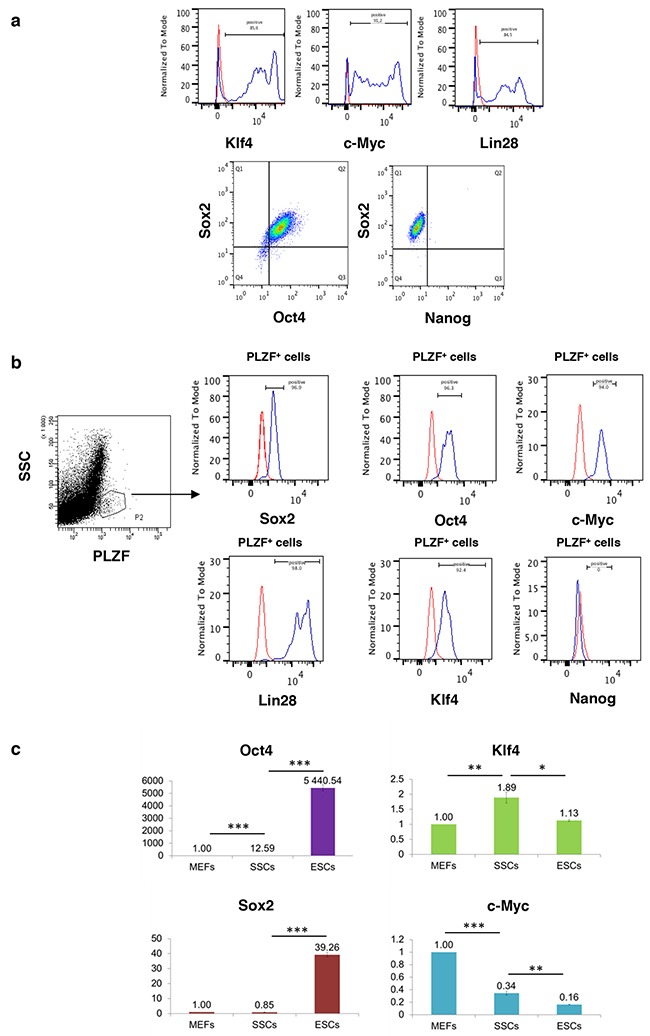
Reprogramming factors that induce pluripotency are expressed in spermatogonial progenitors except Nanog **a**. Klf4, c-Myc, Lin-28, Oct4, Sox2, and Nanog expression measured by flow cytometry in cultured SSCs and **b**. *in vivo* in PLZF^+^ undifferentiated spermatogonia. Red, negative IgG control; blue, cells of interest. Quadrants were placed according to IgG controls. **c**. Expression of reprogramming factors in cultured SSCs compared with MEFs and mouse embryonic stem cells (ESCs) by quantitative RT-PCR. The expression levels of SSCs and ESCs are normalized to that of MEFs. Error bars represent SEM, n=3. * P< 0.05, ** P< 0.01, *** P< 0.001.

Due to the role of the OSKM transcription factors [14, 15] or the Oct4, Sox2, Lin28 and Nanog transcription factors [16] in the reprogramming of somatic cells into pluripotent stem cells, we first analysed their expression *in vitro* in SSCs cultures. All of these transcription factors except Nanog were expressed in cultured SSCs (Figure 1a). When testicular cell suspension from adult mice were analysed by flow cytometry to study the co-expression of these transcriptions factors with the marker PLZF using specific antibodies, the same profile of expression was found in the PLZF^+^ undifferentiated spermatogonia population from adult mice, which contain SSCs (Figure 1b). If Oct3/4 and Sox2 mRNA are significantly less detected in cultured SSCs compared with ES cells, then they are more commonly expressed than in mouse embryonic fibroblasts (MEFs) (Figure 1c). In contrast, the KLF4 and c-Myc expression levels are higher in SSCs compared with ES cells. Therefore, OSKM and lin-28 could be involved in the spontaneous reprogramming observed in cultured SSCs as previously mentioned [17], although their expression levels appear to be quite different compared with ES cells.

### Forced expression of OSKM in SSCs and spermatogonial progenitors

**Figure 2:**
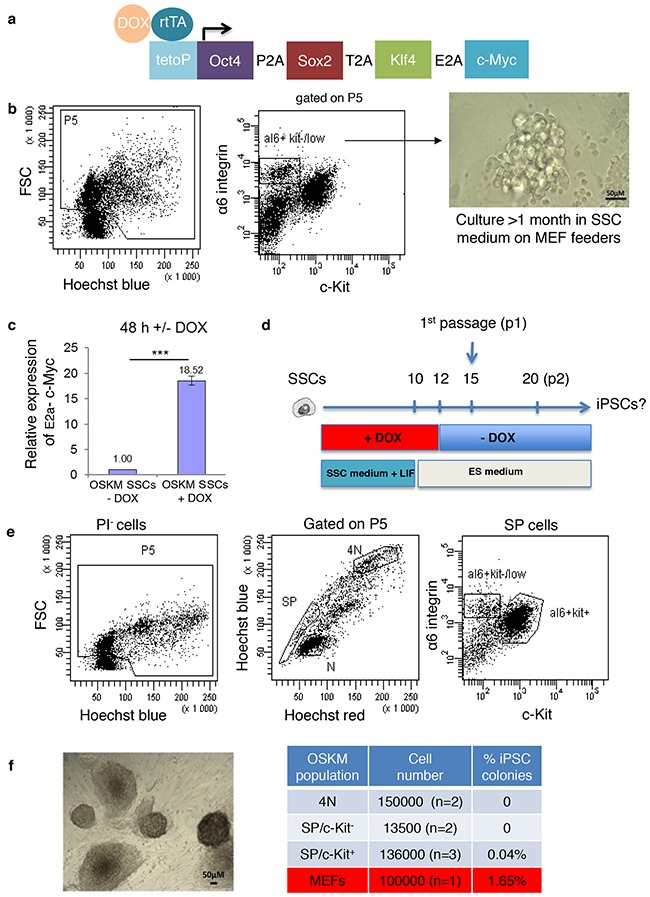
Effects of the overexpression of reprogramming factors after doxycycline induction **a**. Diagram of the single doxycycline-inducible polycistronic transgene in the *Col1a1* locus, which drives the expression of the four mouse reprogramming factors (*OSKM*, encoding Oct4, Sox2, Klf4 and c-Myc) and **b**. the establishment of a long-term culture of SSCs from α-6^+^/c-Kit^-/low^ testicular cells of Col1a1-4F2A-OSKM mice. **c**. Expression of the transgene (E2A-c-Myc, end part of the insert) in established SSCs cultures from Col1a1-4F2A-OSKM SSCs after 2 days of DOX induction (normalized to uninduced SSCs culture, n=3). *** P<0.001. **d**. Protocol used for cell reprogramming. **e**. Sorting strategy of testicular cell populations: spermatogonial populations of SP/α6^+^/c-Kit^-/low^ and SP/ α6^+^/c-Kit^+^ progenitors and the spermatocyte I (4N) population. **f**. Bright-field microscopy showing representative pluripotent colonies obtained after induction using reprogramming factors in a SP/ α6^+^/c-Kit^+^ sorted population, and the reprogramming efficiency of the different cell populations tested.

The expression of OSKM factors has also been described in PGCs, and the forced expression of these factors greatly enhances their reprogramming efficiency [11]. Because the expression levels of Yamanaka's factors appeared to be quite heterogeneous in SSCs and could be insufficient to favour reprogramming, we investigated the effects of the overexpression of these factors on the reprogramming of SSCs into a pluripotent state. We took advantage of the DOX-inducible transgenic Col1a1-4F2A-OSKM mouse model, which was previously developed to reprogram mouse somatic cells and allows the forced expression of the four transcription factors (Figure 2a) [13]. First, a culture of SSCs from homozygous Col1a1-4F2A-OSKM adult mice was established starting from α6^+^/c-Kit^-/low^ testicular cells that were sorted by flow cytometry (Figure 2b). In cultured Col1a1-4F2A-OSKM SSCs (at least 1.5 month of culture, > 10 passages), increased expression of E2A-c-Myc, the end portion of the transgene insert (Figure 2a), confirmed the efficiency of the transgene expression after 2 days of DOX induction (Figure 2c). Then, a reprogramming protocol was applied to the Col1a1-4F2A-OSKM SSCs in the presence of DOX in the 12 (to 18) days before transfer in ES medium without DOX to stop the transgene expression (Figure 2d). We did not generate any pluripotent colony in these conditions (over 1.365 × 10^6^ SSCs tested, n=26 assays). However, 1650 iPSC colonies (1.65%) were generated from 100,000 fibroblasts when the same protocol was applied to mouse embryonic fibroblasts from Col1a1-4F2A-OSKM mice (Figure 2f), which confirmed the great efficiency in reprogramming somatic cells using this model.

**Figure 3:**
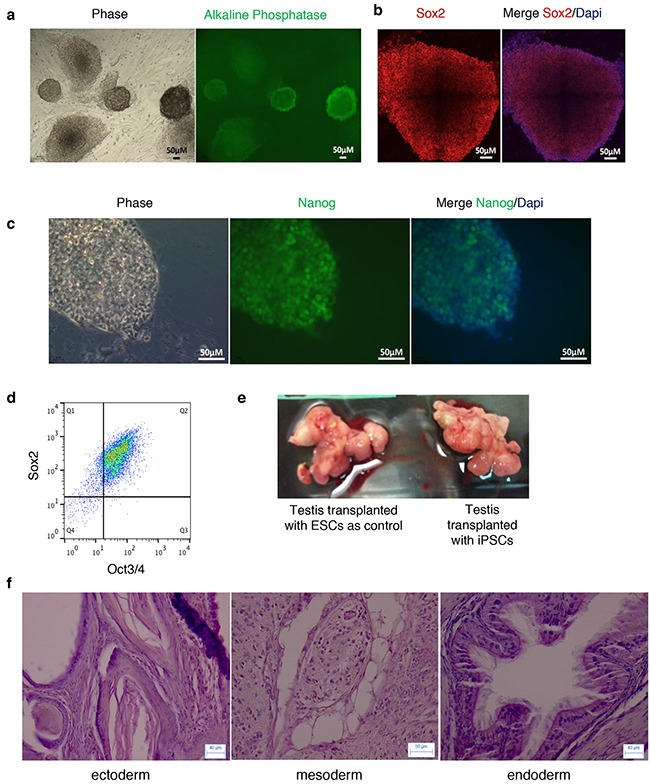
Pluripotency of reprogrammed colonies obtained from SP α6+/c-Kit+ cells **a**. Alkaline phosphatase activity in colonies with an ESC-like morphology analyzed by immunocytochemistry. **b**. Sox2 and **c**. Nanog expression in ESC-like colonies by immunofluorescence. Dapi (blue) nuclear staining. **d**. Cells from the reprogrammed colonies co-express Oct3/4 and Sox2 as shown by flow cytometry analysis. **e**. Teratomas were generated in testis of NSG mice after transplantation of ESCs as a control (left) or with iPSCs from SP α6^+^/c-Kit^+^ reprogrammed colonies (right). **f**. Representative images of sections of teratoma obtained from iPSCs and stained with haematoxylin and eosin showing tissues derived from the three germ layers.

In a second set of experiments, we aimed to study the effects of the forced expression of OSKM factors in SSCs and progenitors that were directly purified from the testes of adult Col1a1-4F2A-OSKM mice. Using a combination of Side Population (SP), α6-integrin, the tyrosine kinase receptor c-Kit, and DNA content, we purified different sub-populations of testicular cells by flow cytometry as previously described (Figure 2e) [18–20]. When these cells were seeded in the presence of DOX, no pluripotent cell colonies were obtained from spermatocytes I (4N) or SP/α6^+^/c-Kit^-/low^ spermatogonial populations, and a few (0.04%) were generated from SP/α6^+^/c-Kit^+^ spermatogonial progenitors (Figure 2f). However, the reprogramming efficiency in the SP/α6^+^/c-Kit^+^ cell fraction was very low compared with the population of MEFs (0.04% versus 1.65%, respectively, Figure 2f). The pluripotency of the reprogrammed cell colonies from the SP/α6^+^/c-Kit^+^ cell fraction was confirmed. These cells had alkaline phosphatase activity characteristic of pluripotent stem cells (Figure 3a), and expressed pluripotent markers such as Sox2 (Figure 3b), Nanog (Figure 3c), and co-expressed pluripotent Oct3/4 and Sox2 markers (Figure 3d). To demonstrate the pluripotency *in vivo*, cells were injected into the testis capsule of immunodeficient NOD *scid* gamma (NSG) mice. Teratoma formed 6–8 weeks later, (Figure 3e) and tumors contained tissues derived from ectoderm, mesoderm, and endoderm (Figure 3f).

**Figure 4:**
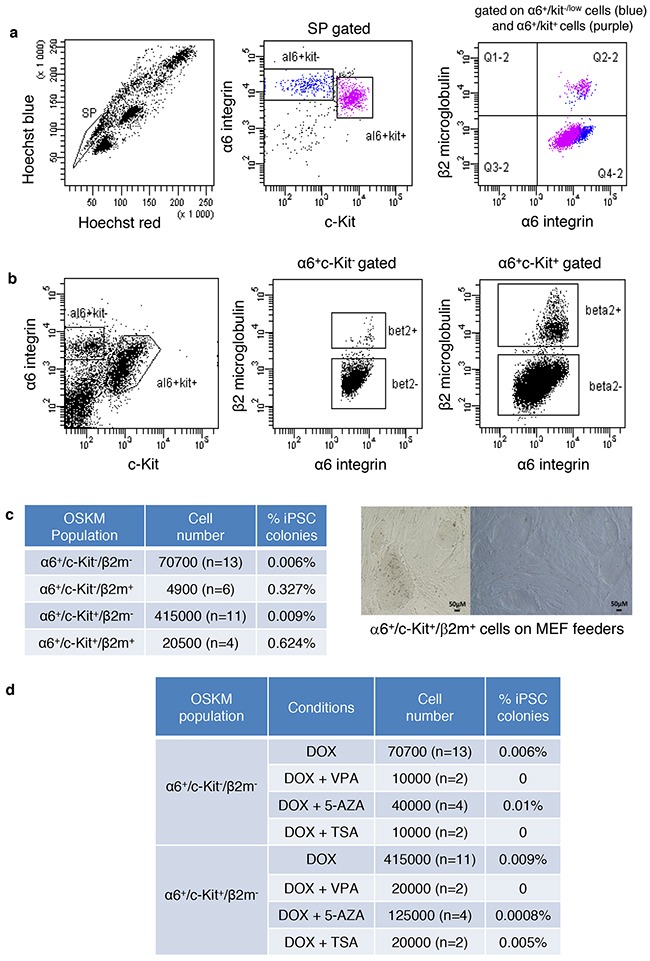
The pluripotent colonies obtained from the SP/α6+/c-Kit+ population originate from contaminating somatic cells **a**. Residual contamination by somatic cells occur in the sorted spermatogonial fractions. SP^+^/α6^+^/c-Kit^-/low^ and SP/α6^+^/c-Kit^+^ populations were discriminated according to β2-microglobulin (β2m), a somatic marker. **b**. α6^+^/c-Kit^+^ and α6^+^/c-Kit^-/low^ cell populations discriminated according to β–2 microglobulin. **c**. Reprogramming efficiency of the different sorted cell populations α6^+^/c-Kit^+^/β2m^-^, α6^+^/c-Kit^+^/β2m^+^, α6^+^/c-Kit^-/low^/β2m^-^, and α6^+^/c-Kit^-/low^/β2m^+^, and bright-field microscopy showing representative images of reprogrammed colonies obtained from the α6^+^/c-Kit^+^/β2m^+^ cell population. **d**. The addition of small molecules does not improve the reprogramming efficiency of sorted germinal cells from transgenic Col1a1-4F2A-OSKM mice. VPA: Valproic acid, 5-AZA: 5-Azacytidine, TSA: Trichostatin A.

Because residual contamination by somatic cells could occur in the sorted spermatogonial fractions, which might contribute to the observed events of reprogramming, we discriminated testicular cell fractions according to β2-microglobulin (β2m), a somatic marker (Figure 4a and Supplementary Figure 1). In addition, we evaluated the α6^+^/c-Kit^-/low^ and α6^+^/c-Kit^+^ populations without selection by the SP marker to consider a larger population of spermatogonial cells. We observed that β2m^+^ cells accounted for 1.37±0.61% and 3.4±1.15% of the α6^+^/c-Kit^-/low^ and the α6^+^/c-Kit^+^ populations, respectively, from the α6^+^ immunomagnetic-enriched testicular cell fraction (Figure 4b). The α6^+^/c-Kit^-/low^/β2m^-^, α6^+^/c-Kit^-/low^/β2m^+^, α6^+^/c-Kit^+^/β2m^-^ and α6^+^/c-Kit^+^/β2m^+^ populations were sorted from the α6^+^ immunomagnetic-enriched cell fraction (n=3) or the total testicular cells (n=2) and underwent the reprogramming protocol. The α6^+^/c-Kit^-/low^/β2m^-^ and α6^+^/c-Kit^+^/β2m^-^ populations did not succeed in generating efficiently pluripotent colonies (Figure 4c), although the expression of the transgene was induced in these populations (respectively 45.8±5.5 fold and 7.8±0.9 fold increase of *E2a-c-Myc* expression in α6^+^/c-kit^-^/low/β2m^-^ and α6^+^/c-kit^+^/β2m^-^ cell fractions 18h after DOX induction). However, iPSC colonies were generated efficiently from somatic α6^+^/c-Kit^+^/β2m^+^ cells (0.624% of 20,500 sorted cells) and α6^+^/c-Kit^-/low^/β2m^+^ cells (0.327% colonies from 4900 sorted cells) with a reprogramming frequency similar to that found for MEFs.

Thus, the somatic cell component appeared to contribute to the reprogramming events observed in the c-Kit^+^ population that were sorted in the first set of experiments. Our results suggest that the reprogramming efficiency by Yamanaka's factors is high in somatic cells but is very low or even non-existent for adult SSCs and progenitors. Together, these results suggest that there must be a specific mechanism in adult germinal cells that prevents reprogramming to a pluripotent state by Yamanaka's factors.

### Combining small molecules or hypoxia with OSKM overexpression does not favour the induction of pluripotent cell colonies in SSCs and progenitors

**Figure 5:**
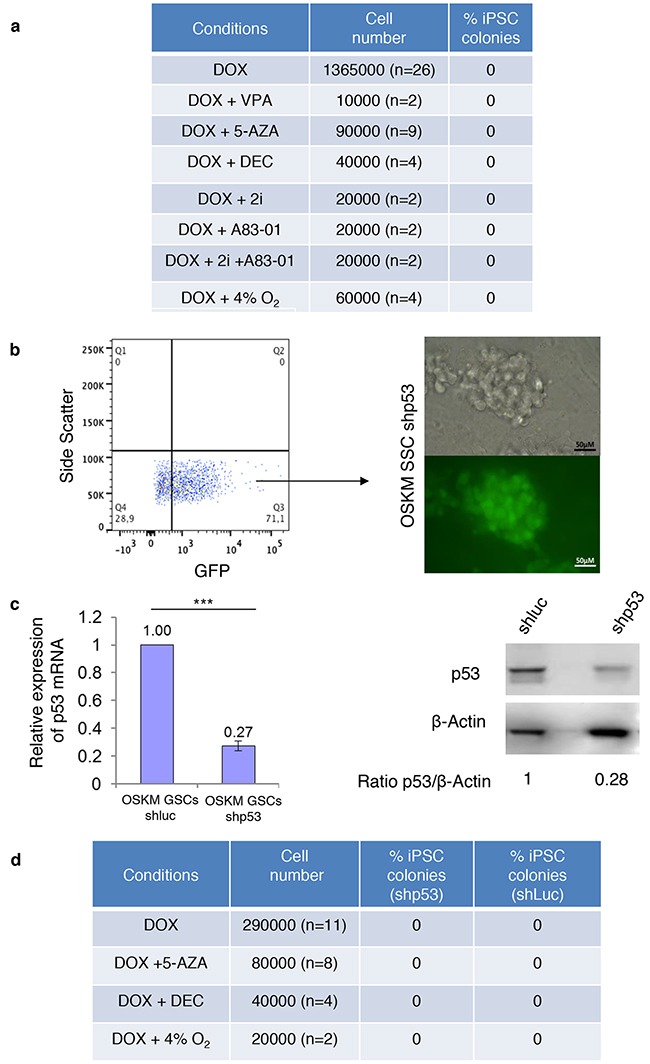
SSCs cultures are not prone to reprogramming even in the presence of small molecules or when p53 activity is diminished **a**. The addition of small molecules does not increase reprogramming efficiency. **b**. Sorting of GFP^+^ cells (quadrants placed according to untransduced cells) and representative images of the culture of Col1a1-4F2A-OSKM-shRNAp53 SSCs obtained after the transduction of Col1a1-4F2A-OSKM SSCs with p53 shRNA-GFP vector (or luciferase shRNA – GFP vector as a control). **c**. Quantitative RT-PCR and Western blot showed a decrease in p53 expression in Col1a1-4F2A-OSKM shRNA p53 SSCs. β-actin was used as a loading control for the Western blots. Error bars represent SEM, n=3. *** P<0.001. **d**. Efficiency of Col1a1-4F2A-OSKM shRNA p53 reprogramming of SSCs using DOX alone or in combination with 5-AZA (2 μM), decitabine (2 μM) or hypoxia.

Several strategies increase the efficiency of the reprogramming of somatic cells into iPSCs. Small molecules, such as 5-azacytidine (5-AZA) and decitabine (DEC), which are DNA methyltransferase inhibitors, and valproic acid (VPA) and trichostatin A (TSA), which are histone deacetylase inhibitors, have been used [21, 22]. First, α6^+^/c-Kit^-/low^/β2m^-^ and α6^+^/c-Kit^+^/β2m^-^ spermatogonial cell fractions were sorted from Col1a1-4F2A-OSKM mice, and 5-AZA, decitabine, VPA, or TSA was added to the cells along with DOX for reprogramming. We did not observe any significant increase in the reprogramming efficiency in α6^+^/c-Kit^-/low^/β2m^-^ or α6^+^/c-Kit^+^/β2m^-^ cells (Figure 4d). Then, we used the cultured Col1a1-4F2A-OSKM SSCs to screen for conditions that could improve reprogramming after DOX induction, including hypoxia culture [23] or other small molecules, such as A-83-01 (a TGFβ signalling pathway inhibitor), PD0325901 (a MAPK/ERK signalling pathway inhibitor), and CHIR99021 (a Glycogen Synthase Kinase 3β inhibitor) [24]. These conditions were still unsuccessful for the generation of iPSC colonies (Figure 5a), and some, such as VPA and 2i, were deleterious for SSC culture (data not shown). Thus, the mechanism that prevents the induced reprogramming of SSCs and spermatogonial progenitors by OSKM cannot be alleviated by specific conditions that were previously described to improve the reprogramming of somatic cells into iPSCs, supporting the hypothesis that the reprogramming mechanisms clearly differ between somatic and germinal cells.

### p53 is not involved in the inefficiency of reprogramming in SSCs and spermatogonial progenitors

Among the roadblocks that can hinder the reprogramming of somatic cells to pluripotency [25] [26], the p53 tumour suppressor pathway could play a role in the repression of reprogramming by OSKM factors in a germinal lineage. The spontaneous appearance of germinal pluripotent colonies was previously reported to occur stochastically in postnatal cultured SSCs [2]. First, we used cyclic pifithrin α, a chemical compound that inhibits p53 function, to improve the reprogramming efficiency of sorted α6^+^/c-Kit^-/low^/β2m^-^ and α6^+^/c-Kit^+^/β2m^-^ spermatogonial cell fractions or cultured SSCs from Col1a1-4F2A-OSKM mice. The presence of pifithrin α or cyclic pifithrin α along with DOX did not significantly change the very low reprogramming efficiency or the lack of reprogramming of these populations (data not shown).

Then, p53 expression was knocked down in cultured Col1a1-4F2A-OSKM SSCs by transducing cells with lentiviral vectors expressing shRNA p53 and GFP. GFP^+-^-transduced cells were purified 48 h after transduction and seeded for cell expansion to obtain Col1a1-4F2A-OSKM-shp53 (Figure 5b). Quantitative RT-PCR and Western blot confirmed the decrease of p53 expression in Col1a1-4F2A-OSKM-shp53 SSCs compared with Col1a1-4F2A-OSKM-shluc control SSCs (Figure 5c). We tried to reprogram Col1a1-4F2A-OSKM-shp53 SSCs in the presence of DOX with or without 5-azacytidine or decitabine and in hypoxia. However, we did not observe any reprogramming events in these conditions (Figure 5d). Taken together, these results emphasize the lack of involvement of p53 in the inhibition of the reprogramming of SSCs by OSKM factors.

In conclusion, we found that the reprogramming efficiency by Yamanaka's factors is high in somatic cells and, notably, β2m^+^ testicular somatic cells, but is negligible or close to zero in adult SSCs. Together with previous data that show that these factors do not induce pluripotency in GS culture from neonatal SSCs [12], our data suggest that postnatal SSCs are resistant to reprogramming by OSKM factors. This finding is quite surprising because almost all cell types are thought to be permissive to OSKM factors [27]. Paradoxically, postnatal SSCs are resistant to reprogramming by OSKM factors, although the germinal lineage is more likely than somatic tissues to spontaneously convert to pluripotency [27]. SSCs might have developed specific mechanisms that repress reprogramming to a pluripotent state by these factors, likely because they are expressed endogenously in SSCs. Despite the role of p53 in the reprogramming of somatic cells [25] and neonatal GS cells from the testis with DMRT1 knockdown [9], the p53 pathway seems to have no role in the prevention of OSKM reprogramming in SSCs. OSKM induction is still working in PGCs at E.11.5 [11], which suggests that epigenetic changes that begin in PGCs when they reach the foetal gonads could help inhibit the reprogramming of postnatal SSCs. Accordingly, knockdown of the DNA methyltransferase Dnmt1 seems to favour reprogramming to pluripotency in p53-deficient neonatal SSCs, even if Dnmt3a/Dnmt3b or Dnmt3l are not involved in this conversion [28]. Thus, epigenetic regulators could be involved in the repression of reprogramming by Yamanaka's factors, even if the chemical molecules that globally modulate the epigenetic status of adult SSCs did not succeed in inducing pluripotency when we forced the expression of OSKM factors in adult SSCs. However, fine tuning the epigenetic regulation of the specific regulatory elements involved in reprogramming could play a role in the repression observed in SSCs.

Differentiation defects of primordial germ cells occurring in fetal life are thought to be at the origin of some testicular cancer. This would be linked to the persistence of immature fetal germ cells in postnatal testis, so called delayed gonocytes still having a pluripotent potential and being potentially tumor-initiating cells [7]. However, the capacity of postnatal germ cells to reprogram is also thought to play a role in germ cell tumour initiation, as for other tissues [29]. First, the emergence of cancer stem cells could originate from a process of dedifferentiation of progenitors, consequent to a mutation or to a deregulated testicular niche abnormally controlling their fate. Second, mutations in SSCs could generate cancer stem cells. Recent works suggest a potential role of OSKM factors in the reprogramming process leading to cancer initiation. *In vivo* reprogramming of somatic cells resulting in the formation of teratoma was described using OSKM reprogrammable mice [30]. In addition, premature arrest in the process of reprogramming by Yamanaka's factor can lead *in vivo* in somatic tissue to the emergence of non pluripotent cancer cells and to cancer development [31]. The primary goal of our work was to address the ability of SSCs/spermatogonial progenitors to reprogram *in vitro* in pluripotent cells in conditions similar to ones used to produce iPS from somatic cells. But, these results can also be viewed with respect to the role of Yamanaka factors in generating cancer-initiating cells in germinal cells. Our data do not support a role for OSKM overexpression in the formation of germinal cancer-initiating cells, and suggest that specific unknown mechanisms prevent this germinal reprogramming by OSKM factors at least *in vitro*. These mechanisms should be alleviated concomitantly with abnormal overexpression of OSKM, in order that OSKM factors induce reprogramming to potential cancer-initiating cells. Even if our experimental conditions do not mimic the natural testicular niche which is more complex, they recapitulate partly the components of this niche as they allow to sustain and amplify spermatogonial progenitors cells at long term, including functional SSCs [18, 32, 33]. Nevertheless, we cannot rule out that OSKM factors combined to other altered molecular pathways could be involved *in vivo* in the process of germinal oncogenesis, or that reprogramming of spermatogonial stem cells and progenitors in postnatal testis could also be preferentially induced by other yet unknown combination of transcription factors, potentially generating cancer-initiating cells. In addition, the reprogramming efficiency of the testicular somatic cell fraction compared to the germinal one could lead to question the view that teratoma in testis is exclusively derived from germinal cell lineage. Further studies are necessary to evaluate the respective roles of the different hypothesis in the generation of germinal cancer-initiating cells, namely remnant embryonic cells in adult tissue and adult spermatogonial cell reprogramming. Deciphering the mechanisms responsible for the inhibition of the reprogramming by OSKM factors in SSCs should more generally benefit the understanding of the reprogramming events observed in the conversion of somatic cells to iPSCs, but also help define the molecular events involved in the initiation of germ cell cancer in testis.

## MATERIALS AND METHODS

### Mice

The reprogrammable Gt(ROSA)26Sor^tm1(rtTA*M2)Jae^Col1a1^tm3(tetO-Pou5f1,-Sox2,-Klf4,-Myc)Jae^/J mice (Col1a1-4F2A-OSKM) [13], which were purchased from the Jackson Laboratory, and the immunodeficient NSG mice were housed in our animal facility. Experiments were performed in compliance with European legislation and the Ethics Committee of the French Ministry of Agriculture (Agreement B9203202).

### Testicular single-cell suspensions, immunomagnetic and flow cell sorting, and flow cytometry analysis

Testicular single-cell suspensions were prepared from 2–3-month-old Col1a1-4F2A-OSKM mice as described previously [34]. The immunomagnetic selection of α6^+^ cells was performed using anti-α6 integrin-PE (GoH3) (BD Pharmingen) and anti-PE microbeads (Miltenyi Biotec) according to the manufacturer's protocol. Hoechst staining (5 μg/ml) of the cell suspensions was performed as described previously [34]. Cells were then labelled with β2m-FITC (Santa Cruz) and anti-CD117-APC (2B8) antibodies (BD Pharmingen). Propidium iodide (Sigma) was added before cell sorting to exclude dead cells. To analyse the transcription factors, cells were fixed and permeabilized using a Cytofix/Cytoperm kit (BD Pharmingen) and stained with the following antibodies: Alexa Fluor 488 mouse anti-Nanog (BD Pharmingen 560261 clone M55-312), Alexa Fluor 647 mouse anti-Sox2 (BD Pharmingen 560294 Clone 245610), rabbit anti-c-Myc (Cell Signaling), rabbit anti-Kfl4 (Abcam), rabbit anti-Lin28 (Cell Signaling), rabbit IgG isotype control (Cell Signaling) and secondary anti-rabbit Alexa Fluor 488 antibody along with APC-anti-PLZF antibody (R&D). Analyses and cell sorting were performed on ARIA, LSR II and FACSCalibur flow cytometers (Becton Dickinson).

### SSCs and MEF culture

To derive adult SSCs lines from adult C57BL/6 and Col1a1-4F2A-OSKM mice, α6^+^/c-Kit^-/low^/β2m^-^ germinal cells were isolated and maintained on mitomycin-C-treated MEFs as previously described [18]. The SSCs culture medium was composed of Stem Span (Stemcell Technologies) and B27 supplement (Life Technologies) and supplemented with recombinant human GDNF (40 ng·ml^-1^, R&D Systems), recombinant rat GFRα1 (300 ng·ml^-1^, R&D Systems), FGF2 (1 ng·ml^-1^, Life Technologies), and ES-Cult™ Foetal Bovine Serum (1%, Stemcell Technologies). Every 3-4 days, SSCs clusters were split using enzymatic digestion with 0.05% trypsin-EDTA (Life Technologies). SSCs were used for reprogramming after at least 1.5 months of culture. MEF cultures were established by trypsin digestion of Col1a1-4F2A-OSKM 13.5 dpc embryos, and the resulting cells were cultured in DMEM supplemented with 10% FBS, L-glutamine and penicillin/streptomycin.

### Reprogramming

The testicular-sorted cell fractions or SSCs lines from adult Col1a1-4F2A-OSKM male mice were cultured on mitomycin-C-treated MEFs with SSCs culture medium supplemented with recombinant mouse LIF (1000 u/ml, Millipore) and 2 μg ml^−^^1^ DOX to induce OSKM factors for 10-18 days. Then, the medium was replaced with ESC culture medium in the presence of DOX for 2 days. The pluripotent colonies were expanded after colonies with ES cell morphology were picked or trypsinized after DOX withdrawal. ESC medium was composed of Dulbecco's modified Eagle's medium (DMEM) (Life Technologies) supplemented with Knockout Serum Replacement (20%, Life Technologies), glutamine (1%, Life Technologies), MEM Non-Essential Amino Acids (1%, Life Technologies), penicillin/streptomycin (1%, Life Technologies) and recombinant mouse LIF (1000 u/ml, Millipore). Col1a1-4F2A-OSKM MEFs were seeded on mitomycin-C-treated MEF feeders in ESC medium and induced with DOX for reprogramming. Hypoxia (4% O_2_) was established in a hypoxic Sanyo incubator. The small molecules used (depending on the experiment) and their final concentrations are listed as follows:

**Table:** 

Molecule	Company	Final concentration
Valproic Acid (VPA)	Sigma Aldrich	2 mM
5-azacytidine (5-AZA)	Sigma Aldrich	2 μM
Stemolecule™ PD0325901 (2i)	Stemgent	0.5 μM
Stemolecule™ CHIR99021 (2i)	Stemgent	3 μM
A83-01	Stemgent	0.25 μM
Trichostatin A (TSA)	Sigma Aldrich	20 nM
Cyclic pifithrin α (cPFT α)	Sigma Aldrich	30 nM
Decitabine (DEC)	Tocris	2 μM

### Lentiviral vector production and transduction of cells in SSC culture

Viral particles were produced by transient transfection of 293T cells with the lentiviral vectors pTRIP-shp53-GFP or pTRIP-shLuc-GFP [35] and the packaging plasmids pCMVDR-8.92 and pMD2G. Viral supernatants were collected 48–72 h later. SSCs were infected by exposing SSCs to viral supernatants overnight in SSC medium containing 5 μg·ml^−1^ polybrene. SSCs were then plated on mitomycin-C-treated MEFs and expanded after selection based on GFP fluorescence by cell sorting.

### RNA extraction and quantitative RT-PCR

mRNAs were prepared from MEFs, ESCs and the following established SSC lines: Wild-type (C57BL/6), Col1a1-4F2A-OSKM, Col1a1-4F2A-OSKM shLuc, and Col1a1-4F2A-OSKM shp53 using the RNeasy® Micro and Mini kits (Qiagen); they were reverse-transcribed with Quantitect kit (Qiagen). Quantitative RT-PCR was performed using an AB7900 device (Applied Biosystems), with Fast SYBR® Green Master Mix (Applied Biosystems), except for β-actin and TRP53, which were analysed with TaqMan® Master Mix (Applied Biosystems).

The primers used are listed as follows:

**Table:** 

Gene	Primer	Sequence or Ref.
GAPDH	R	5′-CCCTTTTGGCTCCACCCT-3′
	F	5′-TTCACCACCATGGAGAAGGC-3′
HPRT	R	5′-AAAGGAAATCCAGTGGCGC-3′
	F	5′-GGCTGGAGATGTTGAGAGCAA-3′
oct-04	R	5′-AGAACCATACTCGAACCATCC-3′
	F	5′-ACATCGCCAATCAGCTTGG-3′
SOX2	R	5′-TGGAGTTGTACTGCAGGGCG-3′
	F	5′-ACAGATGCAACCGATGCACC-3′
KLF4	R	5′-CCGTCCCAGTCACAGTGGTAA-3′
	F	5′-GCACACCTGCGAACTCACAC-3′
c-MYC	R	5′-TGCCTCTTCTCCACAGACACC-3′
	F	5′-ACCACCAGCAGCGACTCTGA-3′
E2A-c-MYC	R	5′-AAAGGAAATCCAGTGGCGT-3′
	F	5′-GGCTGGAGATGTTGAGAGCAA-3′
PLZF	R	5′-TTCCCACACAGCAGACAGAAG-3′
	F	5′-CCCAGTTCTCAAAGGAGGARG-3′
NANOS2	R	5′-CCCCTTCAGGGGTCTTCA-3′
	F	5′-GCAACTTCTGCAAGCACAATG-3′
CYP11A1	R	5′-TGCTGGCTTTGAGGAGTGGAACC-3′
	F	5′-AGGGGTGGACACGACCTCCA-3′
CYP17A1	R	5′-GGTCTGTATGGTAGTCAGTATCG-3′
	F	5′-CCAGATGGTGACTCTAGGCCTCTTGTC-3′
αSMA	R	5′-GGAGCCACCGATCCAG-3′
	F	5′-AACGCTTCCGCTGCCC-3′
VASA	R	5′-GAAGGATCGTCTGTCTGAACA-3′
	F	5′-GAAGAAATCCAGAGGTTGGC-3′
STRA8	R	5′-CTAAGCTGTTGGGATTCCCATC-3′
	F	5′-TGAAGCTCAAAGCATCCTTCAA-3′
AMH	R	5′-ACGGTTAGCACCAAATAGCGG-3′
	F	5′-TTGCTGAAGTTCCAAGAGCCTC-3′
HSD3B	R	5′-GGCACACTTGCTTGAACACAG-3′
	F	5′-TGCACAAAGTATTCCGACCAGA-3′
STAR	R	5′-GCGGTCCACAAGTTCTTCAT-3′
	F	5′-GAAAGCCAGCAGGAGAACG-3′
LHCGR	R	5′-CAGGGATTGAAAGCATCTGG-3′
	F	5′-GAGACGCTTTATTCTGCCATCT-3′
β-actin	QuantumRNA™ β-Actin Internal Standards AM1720 (primers and probe, Life Technologies)	
TRP53	Mm01731287_m1 (primers and probe, Life Technologies)	

### Immunohistochemistry

The cells were fixed with 4% paraformaldehyde, blocked and incubated sequentially with primary antibodies and secondary antibodies overnight at 4°C. The antibodies used were Alexa Fluor 488 mouse anti-mouse Nanog (BD Pharmingen 560261 clone M55-312) and Alexa Fluor 6478 mouse anti-Sox2 (BD Pharmingen 560294 Clone 245610).

### Alkaline phosphatase activity

The AP Live Stain kit (Molecular Probe) was used on cultured cells. The culture medium was removed and, after 2 washes, replaced with DMEM supplemented with 500X AP Live Stain solution (1/500 dilution) for 30 minutes at 37°C. After the removal of the solution and 2 washes, DMEM was added to the cells for the time required for fluorescence observation and replaced with culture medium.

### Teratoma assay

Approximately 5.10^5^ to 1.10^6^ reprogrammed cells obtained from Col1a1-4F2A-OSKM male mice or ESCs (as a control) were injected with Matrigel® (Becton Dickinson) in the testis of NOD *scid* gamma (NSG) mice. Six to eight weeks later, the teratomas were surgically removed and embedded in paraffin. Sections were stained with haematoxylin and eosin.

### Western blot

SSCs pellets were dissociated in cold RIPA buffer (Sigma Aldrich) supplemented with 0.3 μl phosphatase inhibitor 100X (Thermo Fischer) and 1.2 μl protease inhibitor 25X (Roche). Samples were then boiled for 10 minutes at 80°C in Laemmli buffer 4X (Life Technologies) supplemented with antioxidant 10X (Life Technologies). Samples (40 μg) were migrated on NUPAGE Bis-Tris 4-12% gel (Life Technologies) and then transferred on a Hybond N+ filter (Amersham) membrane. Membranes were probed with anti-p53 (Cell Signaling, 2524S) and anti-β-actin (Sigma, clone AC74) primary antibodies and HRP-conjugated secondary antibodies (Thermo Scientific). The reactions were performed with Fast Western Blot kits, SuperSignal™ West Femto (Thermo Scientific 35085).

### Statistical analysis

Error bars represent the SEM, and p values were calculated using Student's t distribution (Prism software). Mean ± SEM are indicated.

## SUPPLEMENTARY FIGURE


