# Supplementary material for: Spermatogonial stem cells and progenitors are refractory to reprogramming to pluripotency by the transcription factors Oct3/4, c-Myc, Sox2 and Klf4
Source: Oncotarget. 2016 Dec 28;8(6):10050–63. doi: 10.18632/oncotarget.14327 (PMC5354640; doi:10.18632/oncotarget.14327)
Supplement: Supplementary file 1 [file oncotarget-08-10050-s001.pdf]

Spermatogonial stem cells and progenitors are refractory to reprogramming to pluripotency by the transcription factors *Oct3/4*, c-*Myc*, *Sox2* and *Klf4*

SUPPLEMENTARY FIGURE

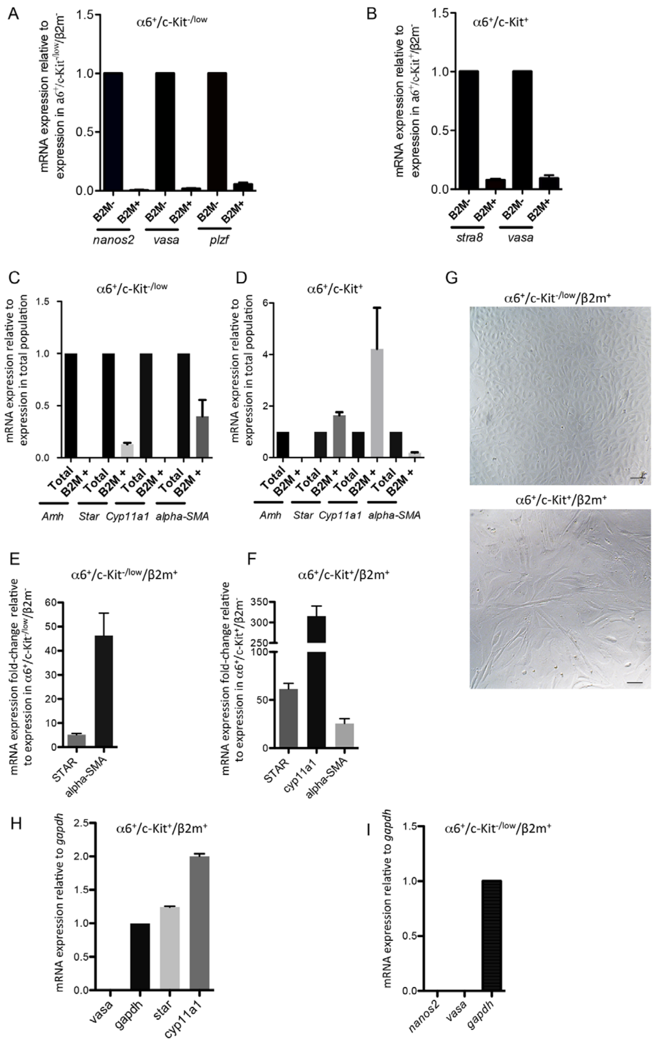

Supplementary Figure 1: Characterization of the sorted testicular cell populations positive for the β2-microglobulin marker. A-B. β2m+ fractions express spermatogonial markers at very low levels compared to β2m- fractions, confirming that β2m+ populations were mainly devoid of germinal cells (n=6, mean ± SEM from two independent experiments) (A) Analysis of α6+/c-Kit-/low/β2m- and α6+/c-Kit-/low/β2m+ populations according to undifferentiated spermatogonial markers *nanos2*, *mouse vasa homolog* and *plzf* (B) Analysis of α6+/c-Kit+/β2m- and α6+/c-Kit+/β2m+ populations according to differentiating spermatogonial markers *stra8* and *mouse vasa homolog*. **C-D.** Analysis of expression of somatic markers *amh*, *star*, *cyp11a1* and α-*SMA* in β2m+ populations compared to expression in total population of testicular cells. (C) α6+/c-Kit-/low/β2m+ express markers of myoid cells (α-*SMA*) (D) α6+/c-Kit+/β2m+ express markers of Leydig cells (*star*, *cyp11a1*). Mean ± SEM of duplicates are indicated. **E-F.** Fold-change of expression of somatic markers in α6+/c-Kit-/low/β2m+ (E) and α6+/c-Kit+/β2m+ populations (F) when compared to respective β2m- populations confirmed that *star*, *cyp11a1* and α-*SMA* expression resides in β2m+ populations (n=5, mean ± SEM from two independent experiments). **G.** Representative pictures of culture starting from α6+/c-Kit-/low/β2m+ and α6+/c-Kit+/β2m+ cells cultivated two weeks without doxycycline and without MEFs feeders. Cultivated cells show clearly morphological traits of somatic cells, bar (20 µm) **H-I.** Expression of markers (Mean ± SEM of triplicates) in cells from culture shown in Supplementary Figure S1G (H) Culture starting from α6+/c-Kit+/β2m+ population express markers of Leydig cells (*star* and *cyp11a1*) and is negative for germinal marker *mouse vasa homolog*, (I) Culture starting from α6+/c-Kit-/low/β2m+ population do not express *mouse vasa homolog* and *nanos2* spermatogonial markers.
